# Ibuprofen sorptive efficacy of zirconium caged date seed derived steam activated alginate beads in a static bed column

**DOI:** 10.1039/d0ra04265j

**Published:** 2020-06-25

**Authors:** Prasenjit Chakraborty, Gopinath Halder

**Affiliations:** Department of Chemical Engg, National Institute of Technology Durgapur Durgapur-713209 West Bengal India gopinathhaldar@gmail.com +91 3432754078 +91 9434788189

## Abstract

The sorption capability of zirconium coupled sodium alginate beads of steam activated biochar derived from date seed (Zr(DSPB)Al) was explored towards Ibuprofen (IBP) removal from simulated water solution in a static bed column. The impact of governing variables *viz.* column bed height (5–25 cm), influent (IBP) concentration (10–30 mg L^−1^) and inflow rate (2–6 mL min^−1^) was investigated in the present study. The column experimentation reflected that with an increase in column bed length, the breakthrough curve height was increased. The maximum sorbent uptake was found to be 23.33 mg g^−1^ from an optimal column bed height of 20 cm, influent (IBP) concentration of 30 mg L^−1^ and inflow rate of 2 mL min^−1^ with the achievement of 94.86% of IBP removal. The bed depth service model (BDST) was studied to examine the sorbent's efficacy and it was observed that column bed height was one of the effective factors towards effective IBP sorption. The Yoon–Nelson model and Thomas model corroborated extremely well with the experimental findings. The desorption study presented a sorbent efficiency up to 5 cycles for IBP exclusion with 37.59% regeneration of the column. The investigation indicated that the novel sorbent Zr(DSPB)Al with proficient performance could be successfully applied for IBP elimination from aqueous solutions.

## Abstract 2

The sorption capability of zirconium coupled sodium alginate beads of steam activated biochar derived from date seed (Zr(DSPB)Al) was explored towards Ibuprofen (IBP) removal from simulated water solution in a static bed column.

## Graphical abstract

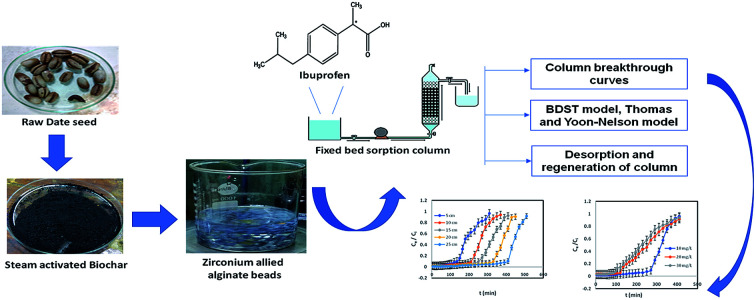

## 1. Introduction

Consequent environmental degradation through pharmaceutical contaminants is considered to be one of the growing scientific concerns in the present day. Enormous numbers of pharmaceutical active compounds are being released into water bodies from various pharmaceutical industries and human usage.^1^ Due to the prevalent consumption of pharmaceuticals, it signifies an increasing fraction of trace growing pollutants in urban aquatic environments.^2^ Increasing global consumption of pharmaceutical compounds shows that the existence of pharmaceutical effluent exclusively non-steroidal anti-inflammatory drugs (NSAIDs) is striking a key anxiety to researchers due to their harmful effects mainly when these components exist as complex mixtures on marine ecosystems and human health.^2,3^ Ibuprofen, one of the chief and vended drugs among other NSAIDs over the globe is often distinguished in water bodies and wastewater sewage.^4,5^ Scientists are concerned with the release of IBP into the biosphere because of its high noxiousness in living cells even at low concentration.^6,7^

Ibuprofen (IBP) has been recognized as an emergent contaminant detected in the treated sewage of wastewater treatment plant.^8^ Ibuprofen (IBP) is widely metabolized to hydroxyl-ibuprofen and carboxy-ibuprofen in water which effects acquisitively in excessive changes in the environment and living organism.^2–9^ The existence of ibuprofen of (0.90–2.11 μg L^−1^) in aquatic bodies have been severally reported.^10^ However, due to long-term biological degradation mechanisms seem to be hardly dynamic for IBP, the exclusion of IBP is an utmost important issue for the environmental safety.^3,11^ Conventional treatment techniques could be partially efficient in order to remove IBP from wastewater treatment plants (WWTP). Hence, the advancement of current treatment plants with more competent technologies is the real task in wastewater treatment to face the developing contaminants.^12,13^ Adsorption technology has appeared as the utmost striking alternative for its easy operation, consistency, and cost efficacy used for liquid and gaseous impurities exclusion.^7,8^

Now a days various studies have been carried out in removing IBP by activated carbon as sorbent.^8^ Numerous adsorbents like raspberry,^14^ graphene oxide nanoplatelets,^15^ potato peel-modified biochar,^16^ olive stone,^13^ modified nano-clay composite,^11^*etc.* have been developed from carbonaceous materials which are the most extensively used for exclusion of IBP from water with high efficacy owing to their immense porosity and admirable surface structure.^7^ The comprehensive practice of the sorption technique is also owing to the prospect of using various kinds of sorbents from waste materials.^3^ The sorption phenomena of IBP onto the activated carbon was due to diffusion of sorbate into the sorbent; and interaction between carboxylic group of IBP and the oxygenated groups (carbonyls and carboxyls group) of the activated carbons.^8^

Date stone (*Phoenix dactylifera*) is accountable for 6–12% of the fruit production in the globe and recognized as one of the prime fruit plants.^17^ Date fruit (*Phoenix dactylifera*) has always been an appreciated crop of mostly desert portions of the world. It can also be treated as one of the most significance crop in agricultural field and growth of the Indian socio economy.^18^ Substantial exploration has been reported on efficacy of date seed derived activated carbon for confiscation of various organic and inorganic contaminants from aqueous media.^19–21^

For wide application in industries, the continuous mode operation is much desirable than the batch mode towards IBP removal from wastewater into a static bed column. Sodium alginate has been used for preparation of immobilized biomass beads for adsorption of various pollutants. The sol–gel formation of sodium alginate is exploited as infrequent component for making an innovative sorbent.^22^ Accordingly, immobilization of sodium alginate was employed effectively in several elements for production of sorbents.^23^ Zirconium allied alginate has been successfully used as novel sorbent for removal of contaminants like fluoride,^24^ Cr(vi),^25^*etc.* from water bodies. However, Zr(iv) linked alginate beads have not been investigated yet as sorbent especially for IBP elimination study in an up-flow column bed. The present exploration investigates the efficacy of date seed derived alginate beads for IBP sorption in column study which substantiates it as an innovative approach to the scientific community towards IBP removal from simulated water. Hence, the novelty of the present research lies in the sorptive riddance of ibuprofen in a static bed column reactor employing zirconium caged date seed derived steam activated alginate beads, the efficacy assessment and the cost-effectiveness of the engineered sorbent towards larger scale application.

Therefore, the rigorous investigations have been carried out in removing IBP from the aquatic system by steam activated date seed biochar confined with zirconium Zr(iv) associated sodium alginate gel. It was reported in various studies that Zr(iv) linked polymers offer higher mechanical strength than other inorganic metal allied beads.^25^ To the best of our knowledge, steam activated sorbent prepared from date seed (*Phoenix dactylifera*) stuck in zirconium allied alginate beads for IBP exclusion has not been testified so far. Sorption mechanism of zirconium alginate and its IBP eradication potentials in an upward flow direction column were observed based on the influent concentration, bed height and inflow rate. The bed depth service time, Thomas and Yoon–Nelson models were explored to investigate the performance of column and interpretation of sorption process on larger scale. The potential ability of the sorbent was confirmed through several desorption–sorption cycles until the column exhaustion stage reached. Cost estimation of Zr(DSPB)Al beads development was also investigated in the present study which is reportedly a significant and innovative incorporation for IBP adsorption in fixed bed column that directs the process economically sustainable. Zirconium confined steam activated date seed biochar alginate bead (Zr(DSPB)Al) as a novel sorbent is expected to produce remarkable efficiency in IBP reduction from waste water in static bed column reactor.

## 2. Experimental investigation

### 2.1. Zr(DSPB)Al beads preparation

The steam activated date seed derived biochar (DSPB) was used for IBP sorption in a static bed column. Initially, date seed (*Phoenix dactylifera*) was rinsed with distilled water after collecting from local market. The washed date seeds were then dried under sunlight for 2 days and for further drying date seed were retained in hot air oven at 60 °C temperature over night to eliminate the entire moistness. Afterwards, dehydrated material was carbonized in a muffle furnace at 700 °C temperature for 1 h. The carbonized biochar was then activated with superheated steam at a pressure of 1.5 kg cm^−2^ and a temperature 800 °C for 1 h with flow rate of 1.2–1.5 kg h^−1^. Finally, physically activated biochar was cooled and placed in desiccator.

DSPB was confined in zirconium ions-based alginate beads. Initially, 5 g of sodium alginate slurry was mixed with 100 mL of distilled water to prepare sodium alginate slurry. Then slurry was continuously stirred for 1 h at 50 °C in a magnetic stirrer to prepare a consistent mixture. Afterwards 1.5 g of DSPB was uniformly added to homogeneous mixture by comprehensive mixing with a speed of 600 rpm. Then 0.1 M Zr solution was prepared from zirconyl oxychloride (ZrOCl_2_) powder for production of beads. The DSPB-alginate gel mixture added drop by drop with the help of medical syringe from 10 cm height in a 0.1 M Zr solution with constant stirred speed of 50 rpm. Then spherical beads were shaped which was stored in Zr solution for 24 h followed by thorough rinsing of beads in deionised water.^25^ Finally, the sorption column was occupied by the produced beads. Hence, zirconium crates steam activated date seed alginate beads Zr(DSPB)Al was stored for IBP exclusion study in column.

### 2.2. Fabrication of column

**Fig. 1:**
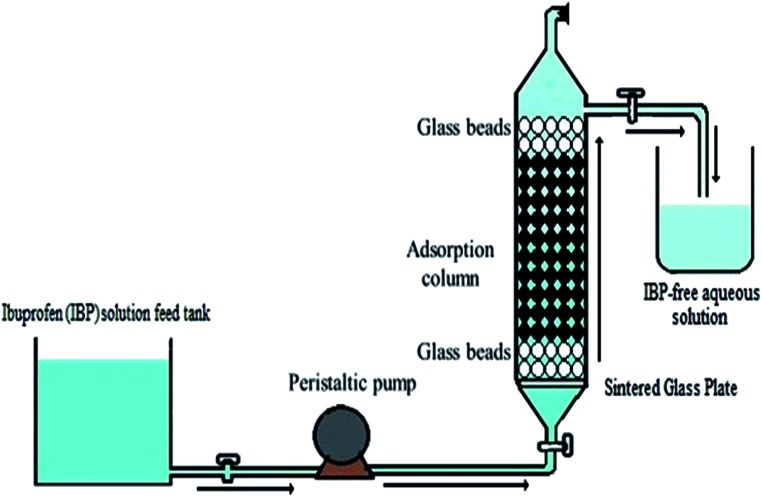
Schematic of static bed up-flow sorption column for IBP exclusion.

An adsorption column was designed considering the maximum holding time for removal of IBP from aqueous phase. Primarily, a glass column of lab scale based was made with design configuration of 2 cm inner diameter and 40 cm of length consisting of IBP solution comprising container, influent channel and an outlet tank. At first the column was rinsed thoroughly with deionized water to discard air bubbles and impurities before beginning of every experiment. GI grade sintered glass were used in the sorption column base. The upper portion of the column was occupied with glass beads to avoid alginate beads floatation. A computed peristaltic pump (WW-73160-31, Cole-Parmer, India) was used for controlling IBP flow rate. For avoiding the plausible variation in the column volume of the effluent was determined at a regular period interval. The upward flow of influent was continued for maximum sorbate–sorbent interface time. The reported studies outlined that the maximum sorption can be attained with greater retention time. So, a comprehensive schematic column diagram was depicted in Fig. 1.

### 2.3. Column experimentation

A simulated solution of ibuprofen was produced in a standard volumetric flask and 1 L of IBP stock solution was used for each column investigation. Three different parameters like influent IBP concentration (10–30 mg L^−1^), bed depth (5–25 cm) and flow rate (2–6 mL min^−1^) were operated with solution pH of 3 in the sorption column. Initially batch mode analysis was performed with blank Zr–Al beads and Zr(DSPB)Al beads to examine the promising role of the beads in the exclusion process. Batch study was executed with 10 mg L^−1^ of initial IBP solution at pH 3 and temperature 20 °C for 24 h under a constant shaking speed of 150 rpm and 1 g of both the beads were used. Optimized results have been obtained at pH 3, initial IBP concentration of 10 mg L^−1^ and time 24 h in batch study for IBP removal from water phase.

### 2.4. Analysis of column design

The performance of static bed column was designated by breakthrough volume (BV) of column which signified as a ratio of *C*_e_/*C*_i_ where, *C*_e_ implies effluent concentration at function time *t* in min and *C*_i_ designates influent concentration in mg L^−1^. Hence, a (*C*_e_/*C*_i_) against *t* plot has been presented for each investigation to measure the column sorption performance.

Several factors like mass transfer length (*Z*_m_) and mass transfer zone (Δ*t*) were also calculated using eqn (1) and (2).^25^

1 Δ*t* = *t*_e_ − *t*_b_

2
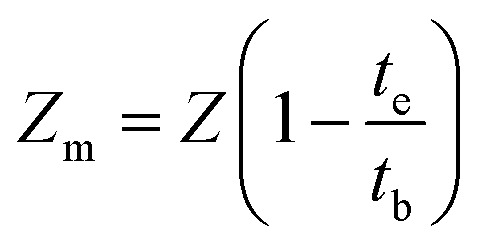
where *t*_e_ signifies exhaustion time of bed and *t*_b_ indicates breakthrough time in min. *Z* signifies the height of bed in cm.

Total column bed sorption capacity (*q*_total_) can be stated in following eqn (3).^25,26^3
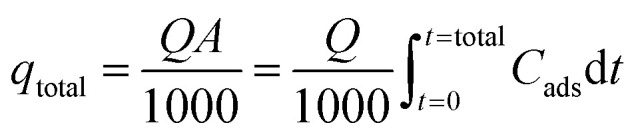
where *Q* symbolizes the flow rate in mL min^−1^ and *C*_ads_ is the variance of *C*_i_ and *C*_e_ for entire time *t*. *A* signifies the area under the breakthrough curve obtained from *C*_ads_*versus t* (min) plot in mg min mL^−1^.

The static column bed sorption capacity at equilibrium condition (*q*_eq(exp)_) is determined by eqn (4) as presented below:^26^4
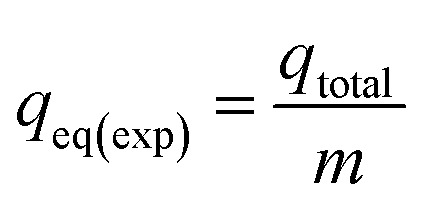
where mass of the sorbent is *m* (g). The subsequent eqn (5) was employed for determining the IBP removal percentage (*R*%) from the column.5
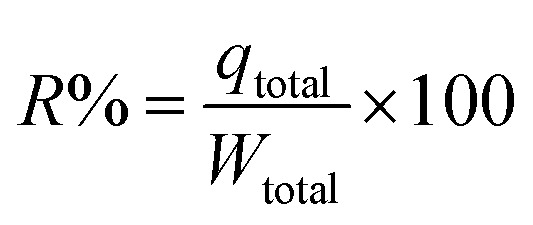
where *W*_total_ was calculated from the eqn (6) as follows6
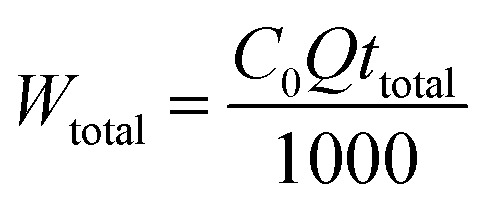


Empty bed retention time (EBRT) is an imperative parameter in the column study. EBRT can be determined using eqn (7).7
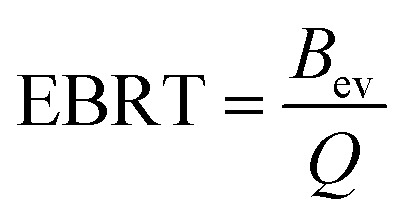
where *B*_ev_ represents volume of empty bed (m^3^), *Q* denotes the discharge (m^3^ min^−1^). AEC, a significant parameter can be determined by the following eqn (8):^27^8
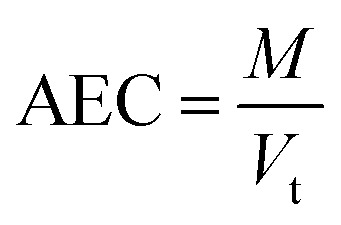
where *M* is the quantity of polluted influent in column and *V*_t_ indicates bed exhausts volume. Again, sorption column re-usability was determined for multiple desorption–sorption cycles. Consequently, total IBP desorption capacity of sorption column was determined by the following eqn (9).^25^9
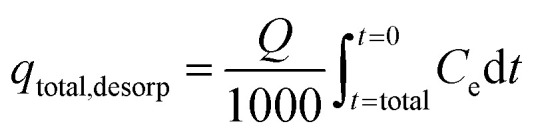


At equilibrium condition, IBP desorption capacity (*q*_eq, desorp_) in mg g^−1^ can be determined by eqn (10).10
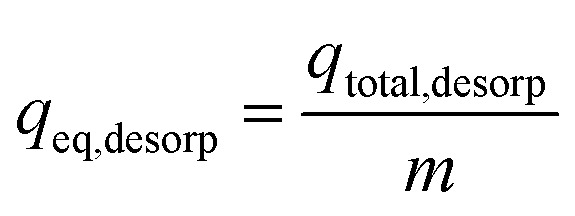


Lastly, eqn (11) was used to calculate the sorption ability of the column after desorption in terms of regeneration percentage.11
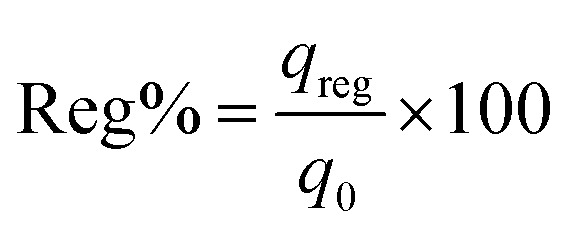
where Reg% signifies the column regeneration percentage after each sorption–desorption cycle and *q*_reg_ denotes sorption capability of spent adsorbent after regeneration and *q*_o_ represents adsorption ability of sorbent in mg g^−1^.

## 3. Results and discussion

### 3.1. Morphological investigation of sorbent (Zr(DSPB)Al)

**Fig. 2:**
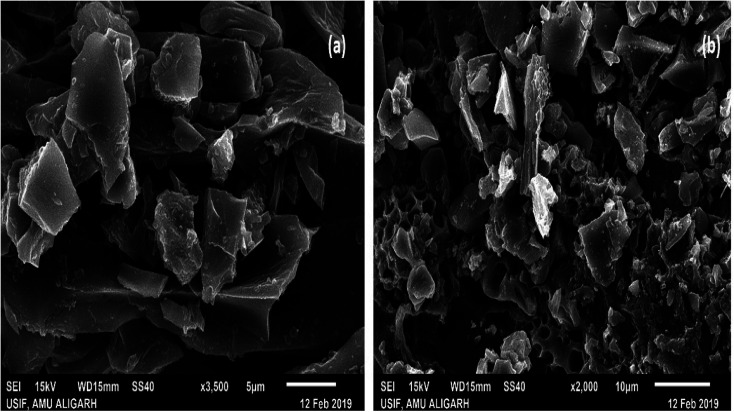
Morphological study of Zr(DSPB)Al (a) raw Zr(DSPB)Al (b) spent Zr(DSPB)Al.

The surface area and pore volume of DSPB was reported to be 513 m^2^ g^−1^ and 0.205 cm^3^ g^−1^ correspondingly in our earlier work.^19^ Surface morphology of Zr(DSPB)Al was inspected through scanning electron microscope (Jeol, JSM-6510, Japan). Initially, a palladium coating (8 nm thick) was used in the produced alginate beads at a rate of 30 mA for 30 s using an auto fine coater to increase the conductivity of the beads as it is a non-conductive material. An infra-red lamp was used for drying of the prepared samples followed by SEM investigation for morphological analysis. Hence, it was found in Fig. 2(a) that alginate beads polymerization by zirconium oxychloride presented an uneven shaped pore before adsorption. Moreover, uneven extended surface was found in the bead exterior for IBP devotion. It was detected that coarse site of the bead surface provides enough active site for adhesion of sorbate. The light greyish layer was found on the pores of the bead surface in Fig. 2(b) which endorsed the IBP adhesion on the surface of the beads. The adsorption of IBP ions onto the sorbent surface may be due to sorption on interface between the sorbate and adsorbents.

### 3.2. Elemental composition of the sorbent

**Fig. 3:**
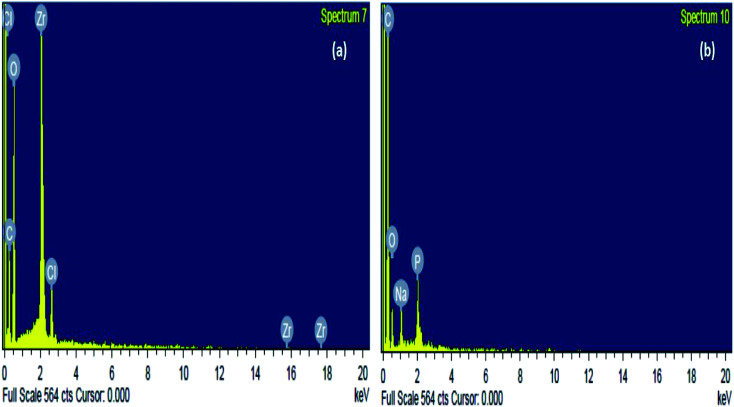
EDX investigation of Zr(DSPB)Al (a) raw Zr(DSPB)Al (b) spent Zr(DSPB)Al.

The elemental composition of the sorbent before and after sorption was inspected through energy-dispersive spectroscopy or EDX analysis. Consequently, it was found from Fig. 3(a) and Table 1 that the elemental constituents of the raw Zr(DSPB)Al were C, O, Zr and Cl. The existence of Zr and Cl may be due to attachment of zirconium oxychloride ion and sodium alginate onto DSPB biochar. After IBP sorption there was a fundamental variation owing to addition of IBP onto the sorbent. In Fig. 3(b) and Table 1, a massive increase of carbon percentage was noticed and presence of Na and P was also observed after IBP adhesion which might be due to attachment of IBP ion and sodium alginate cages biochar.

**Table 1:** EDX investigation of raw and spent Zr(DSPB)Al

Weight% of elemental component of the adsorbent			
Zr(DSPB)Al (raw)		Zr(DSPB)Al (spent)	
Element	Weight%	Element	Weight%
C K	32.51	C K	64.29
O K	44.43	O K	31.72
Zr L	20.85	Na K	3.42
Cl K	2.21	P K	0.57
Total	100.00	Totals	100.00

### 3.3. FTIR study

**Fig. 4:**
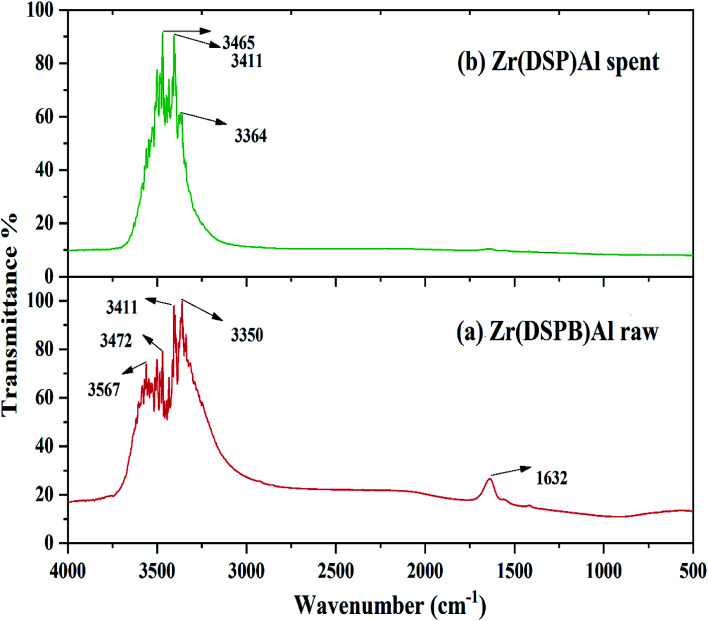
FTIR study of Zr(SABC)Al (a) raw Zr(SABC)Al (b) spent Zr(SABC)Al.

IBP adhesion on to the sorbent exterior mainly depends on the existence of the functional groups on the surface of the adsorbent. Functional group's presence on the sorbent surface before and after IBP adhesion can be predicted by FTIR analysis (PerkinElmer C109292, Thermo Fisher Scientific, India). The surface chemistry of Zr(DSPB)Al has been explored through FT-IR study in the range of 4000–400 cm^−1^. Hence, exploration of Zr(DSPB)Al sorbent before and its alterations after IBP sorption have been presented in Fig. 4(a) and (b) which elucidates numerous groups like alcohol (O–H) stretch at 3567 cm^−1^, 3472 cm^−1^ and 3411 cm^−1^, amine (N–H) stretch at 3350 cm^−1^ and amide (C=O) stretch at 1632 cm^−1^ that were detected on the surface of (Zr(DSPB)Al) before and after adsorption of IBP. The alcohol (O–H) stretch at 3465 cm^−1^ and amine (N–H) stretch at 3364 cm^−1^ were slightly shifted and no such alterations were found in other bands. However, major shifting of peaks were noticed for alcohol (O–H) stretch and amine (N–H) stretch after sorption of IBP owing to existence of carboxylic acid group in ibuprofen which may be attached with alcohol (O–H) and amine (N–H) group confirming IBP sorption in (Zr(DSPB)Al). The alcohol (O–H) stretch and amine (N–H) stretch of sorbent material could be adhered with (C=O) bond in the carboxylic group (–COOH) of IBP. Consequently, FTIR investigation endorses that the maximum sorption resulted due to the presence of alcohol and amine stretch on the active sites of the sorbent surface. It may confirm from the IR band alteration as diffusion of IBP molecules into the pores and active sites of the sorbent.

### 3.4. Possible sorption mechanism of IBP removal study by Zr(DSPB)Al beads

Adsorption mechanism is a significant study pertaining to sorbate adherence phenomena onto the sorbent which represents a substancial reason behind the success of a sorption process. In the current study Zr(DSPB)Al beads were employed for ibuprofen sorption in the column. IBP sorption possibilities on the surface of adsorbent can be illustrated through functional groups present in the sorbent surface. As SEM images are presented in the previous section, it can be seen that IBP sorption occurred at specific active site of the surface of the adsorbent may be owing to the fact that zirconium linked alginate beads were provided a greater exposure to adhesion.^25^ FTIR study designated that surface chemistry of Zr(DSPB)Al beads exhibited a substantial involvement towards sorption of IBP ion. Functional groups like alcohol group and amine group promoted sorption probabilities of IBP on the surface of the Zr(DSPB)Al bead. Presence of carboxylic acid group in ibuprofen may produce bond with alcohol (O–H) and amine (N–H) group in the active sites, and pores of the Zr(DSPB)Al beads can be accountable for the probable adsorption mechanism of the IBP ion and sorbent.

### 3.5. Initial study of blank Zr–Al beads in the IBP removal procedure

**Fig. 5:**
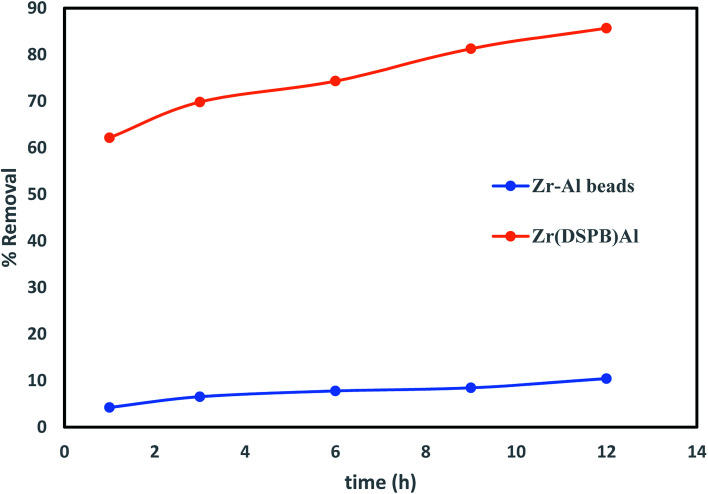
Comparative analysis of IBP removal capability of blank Zr–Al beads.

The efficiency of an immobilized sorbent was examined to excerpt the influence of the immobilizing material in IBP exclusion process. Therefore, blank zirconium alginate beads were verified in Fig. 5 recommends that blank Zr–Al beads displayed 10.44% IBP removal as compared to Zr(DSPB)Al bead presented removal of IBP increased up to 85.7%. This proposes that though Zr–Al contributed in the exclusion process but its effect is less as compared to Zr(DSPB)Al where superheated steam activated biochar was mainly accountable for IBP adsorption.

### 3.6. Parametric assessment for IBP sorption

The performance efficiency of column has been confirmed through breakthrough curve of *C*_*t*_/*C*_0_*vs.* time (*t*) plot in the subsequent section. Implementation of the column sorption method was designated from characteristic breakthrough curve. Therefore, functioning optimization parameters *viz.* column bed height, influent concentration of IBP and inflow rate were explored from breakthrough curve shape where its sharpness reformed consequently under different experimental condition.

#### 3.6.1. Influence of bed height

**Fig. 6:**
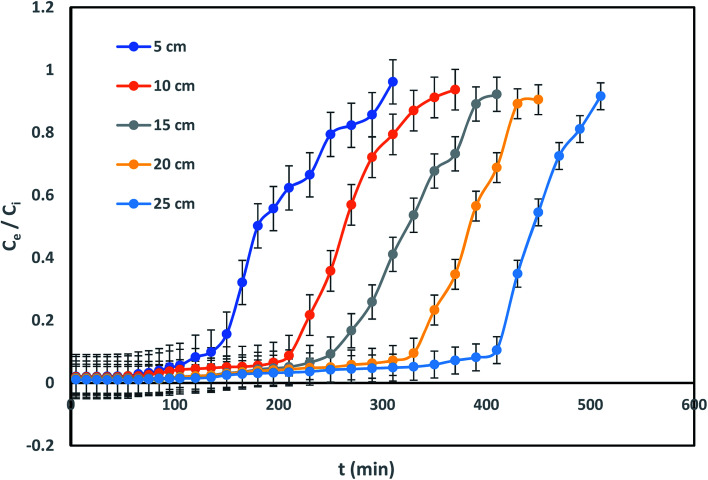
Influence of bed height on IBP sorption.

The effect of bed height towards IBP removal was examined for different bed depth of 5, 10, 15, 20 and 25 cm with constant IBP influent concentration of 10 mg L^−1^ and 2 mL min^−1^ of flow rate. Successive breakthrough curves for various bed heights were demonstrated in Fig. 6. It shows that the breakthrough time is increased with rise in bed length. It was observed that breakthrough point of bed depth 5, 10, 15, 20, and 25 cm was 105, 180, 210, 250, and 310 min correspondingly. Also, the bed exhaustion point for bed height of 5 cm was 310 min and it was increased to 510 min for the bed height of 25 cm. The present variances in equally breakthrough time and bed exhaustion time may be owing to fact of rise in column bed volume increases IBP sorbing sites. Therefore, the quantity of IBP attachment increased with rise in bed height.

However, it can be identified that due to accessibility of better mass-transfer boundary, the breakthrough curve inclines more projecting with increase of each bed height. Similar kind of trend has been reported earlier.^5^ Better exclusion of pollutants from aquatic phase can be attained at greater bed height due to available of greater active sites for devotion of sorbate onto sorbent. Uptake capability also increased progressively with an escalation in bed height as listed in Table 2. Similarly, it can be suggested that rise in sorbent sorption capacity for a longer time since increase in EBRT for each eminent column.

**Table 2:** Column performance indicators for IBP removal by Zr(DSPB)Al

Operational parameters			Column performance indicator							
*C*_i_	*Q*	*Z*	Δ*t*	*Z*_m_	EBRT	AEC	*q*_total_	*q*_ex(exp)_	*W*_total_	*R*%
10	2	5	205	3.30	31.14	2	5.82	4.77	6.2	93.99
10	2	10	190	5.13	62.8	2.29	6.98	4.11	7.4	94.44
10	2	15	180	6.58	94.2	2.68	7.78	3.53	8.2	94.88
10	2	20	180	8	125.6	3	8.54	3.16	9	94.93
10	2	25	160	7.48	157	3.13	9.68	3.02	10.2	94.97
10	2	20	180	8.78	125.6	3.29	7.73	2.86	8.2	94.39
20	2	20	190	14.14	125.6	3.29	15.53	5.75	16.4	94.7
30	2	20	325	15.85	125.6	3.29	23.33	8.64	24.6	94.86
10	2	20	220	11.89	125.6	3.64	6.99	2.59	7.4	94.58
10	4	20	155	14.76	62.8	3.21	7.04	2.60	8.4	83.83
10	6	20	125	16.66	41.86	3	6.78	2.51	9	75.39

#### 3.6.2. Impact of influent (IBP) concentration

**Fig. 7:**
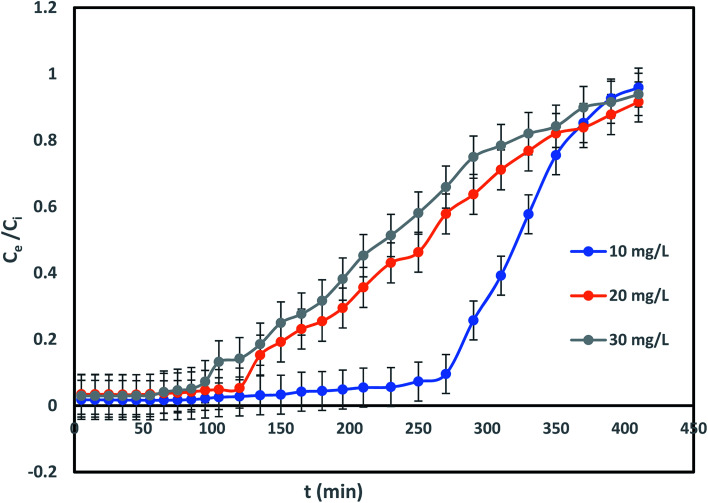
Influence of influent concentration on IBP sorption.

Impact of IBP concentration on its confiscation was determined by changing its concentration from 10 to 30 mg L^−1^ at a flow rate of 2 mL min^−1^ and bed height of 20 cm. It can be shown in Fig. 7 that difference in initial IBP concentration from 10 mg L^−1^ to 30 mg L^−1^ was significantly exaggerated the breakthrough curve. Breakthrough time was perceived to be reduced from 230 min to 80 min with a rise in initial IBP concentration from 10 to 30 mg L^−1^. The delay in breakthrough time in less IBP concentration may due to slow mass transfer occurred in IBP sorption. The IBP adsorption capacity of adsorbent improved from 7.73 mg g^−1^ to 23.33 mg g^−1^ with increasing influent concentration, which can be attributed to the matter that faster saturation of the adsorbent at lower concentration. Fig. 7 displayed the deviation of the curve to left with each IBP concentration increasing, followed by rise in sharpness of the curves at greater IBP concentration. This may be accredited to the point that concentration incline was increased with rise in IBP concentration which resulted reduction in interface of mass-transfer. The exhaustion time of column for 10 to 30 mg L^−1^ of IBP concentration was observed to be almost same about 410 min. Therefore, it may be established that overloading of IBP ions reduced the prospect of surface contact at an initial stage that resulted quicker breakthrough time. The comparable drift has been explored previously.^14^

#### 3.6.3. Effect of inflow rate

**Fig. 8:**
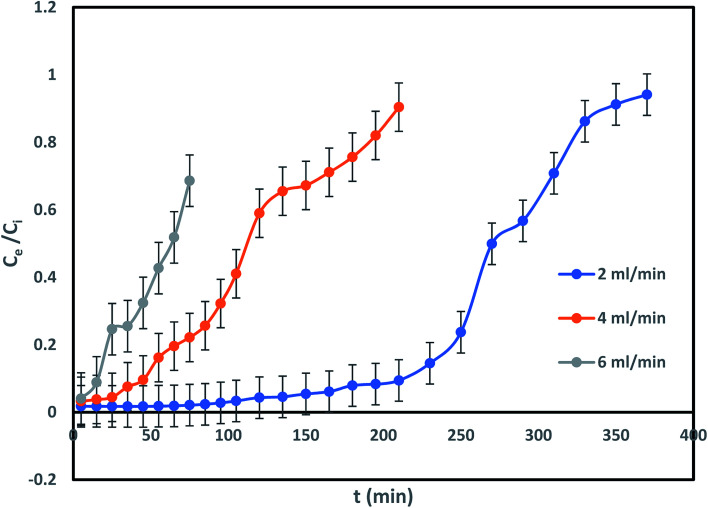
Impact of influent flow rate on to IBP sorption.

Impact of column flow rate in IBP remotion study has been conducted with various inflow rate of 2, 4 and 6 mL min^−1^, fixed influent (IBP) concentration and bed length of 30 mg L^−1^ and 20 cm respectively to inspect the change in the breakthrough curve. Fig. 8 demonstrated that rise in inflow rate reduced the time needed for IBP sorption from aqueous solution which resulted in less sorption occurred at high flow rate. The obtained characteristic parameters from every investigation were presented in Table 2 and Fig. 8. It has been noticed that breakthrough time and exhaustion time of column considerably shrunk from 150 min to 25 min and 370 min to 150 min respectively with rise in influent flow rate from 2 to 6 mL min^−1^. Also, it was observed from Table 2 that the adsorption of IBP got reduced significantly at higher flow rate which may be due to IBP ions getting inadequate time to be diffused onto the sorbent with increasing inflow rate. Therefore, slow inflow rate offers higher residence time for attachment of IBP ions and sorbent exterior facilitating the existence of active sites on the liquid–solid surface. Accordingly, high IBP exclusion can be attained by deferred exhaustion time and lengthy breakthrough time. Similar trend of results for IBP sorption in packed column have been reported in limited studies.^5,14^ Hence, lesser IBP removal occurred at high inflow rate due to availability of less time for external mass transfer and at slow inflow rate lengthier period existing for sorption where intra-particle diffusion controls the sorbate–sorbent interaction in column.

### 3.7. Comparative assessment of IBP removal study in static bed column

The current exploration of IBP removal from simulated solution in continuous method exhibited the favourable outcome as listed in Table 3 in comparison with the few reported studies of sorptive exclusion of IBP in static bed column. The table includes the performance of some different sorbents in removing IBP in a packed column. The Zr(DSPB)Al beads used as sorbent in this study was capable of removing 23.33 mg g^−1^ of IBP ion from synthetic water solution at bed height of 20 cm under a flow rate of 2 mL min^−1^. The sorbent used in the current study presented a substantial result in comparison to other works striking a potential aspect that it is easy to get manufactured with affordable cost. The engineered zirconium associated sodium alginate beads possessed a sufficient mechanical strength which provided its greater sustainability and dimensional retentivity for its multiple uses.

**Table 3:** Comparative study of IBP sorption in the static bed column

Adsorbent	*C*_i_ (mg L^−1^)	*Z*_m_ (cm)	*Q* (mL min^−1^)	*q*_total_ (mg g^−1^)	References
AC-peach stones	10	6.6	2	55.0	4
AC-rice husk	10	7.4	2	22.7	4
Raspberry leaves	10	1.06	1.5	25.98	12
Zr(DSPB)Al beads	30	15.8	2	23.33	Present study

### 3.8. Theoretical interpretation of breakthrough curve

It is essential to design a preliminary process to implement an effective large-scale sorption technology in real practice. Hence, the data acquired from distinct parametric optimization was used in theoretical investigation and larger scale approach towards designing of the separation process. Therefore, three well known models like Bed Depth Service Time (BDST) model, Yoon–Nelson model and Thomas model were employed for mathematical explanation of column designing.

#### 3.8.1. Bed depth service time (BDST) model

**Fig. 9:**
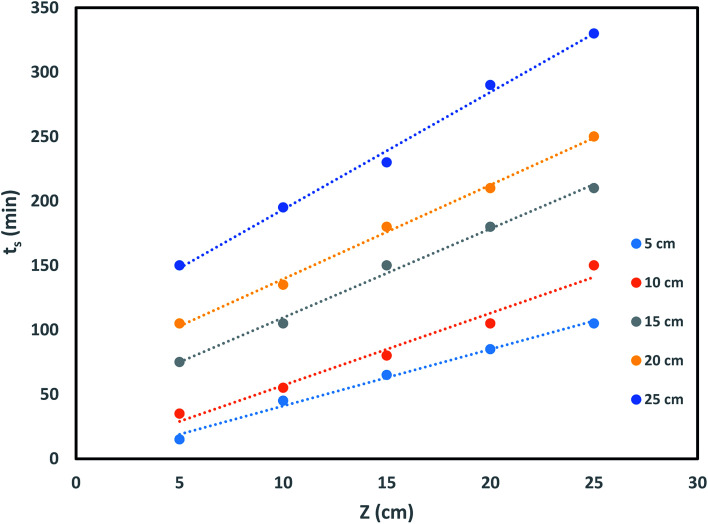
BDST model impact of bed height on IBP sorption.

BDST model is upgraded form of Bohart and Adams (1920) model^28^ and it was used to determine the optimal bed height of a sorption column. Hence, modified BDST model has been presented in the following eqn (12).12
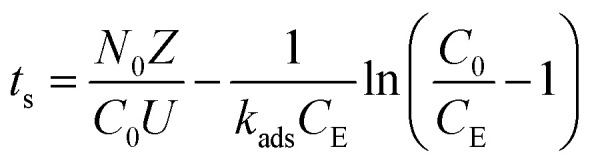
where *t*_s_ signifies time at breakthrough point in h, *N*_o_ denotes active bed capacity in mg g^−1^ and *Z* represents bed depth of the column in cm. *k*_ads_ signifies the sorption rate constant (L mg^−1^ h^−1^). *U* symbolises linear inflow rate in a column (cm h^−1^) and *C*_0_ and *C*_E_ denotes concentration of influent solution and IBP ion concentration at breakthrough point in mg L^−1^ correspondingly. So, from the slope and intercept of straight line of *t*_s_*versus Z* plot as shown in Fig. 9, the others parameters can be measured.

Again, theoretical critical bed depth *Z*_o_ of the column can be determined using eqn (13) which developed at when service time (*t*_s_ = 0) at breakthrough point.13
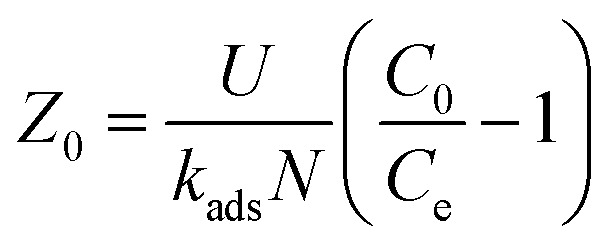


Hence, the effects of bed depth on sorption process in the column and its saturation time and breakthrough time were inspected and theoretical critical bed depth *Z*_o_ was also measured. It may be observed in Table 4 that *N*_0_ and *Z*_o_ values increase with rise in bed depth recommending better sorption in higher bed height of the column. The value of *k*_ads_ as enumerated in Table 4 is moderately better at smaller bed height which proposed that column has the ability of treating pollutant from water bodies for a longer time. Therefore, it may be suggested that column bed height was significant factor leading to the efficacy of the column in sorption process.

**Table 4:** Findings of model parameter of BDST model

Model parameters	Bed depth (cm)				
	5	10	15	20	25
*k*_ads_	0.441	0.261	0.006	0.003	0.002
*N*_o_	6.996	8.904	10.971	11.607	17.29
*R* ^2^	0.991	0.972	0.994	0.996	0.994
*Z*_0_	0.0996	0.395	24.59	64.57	126.05

#### 3.8.2. Thomas model

**Fig. 10:**
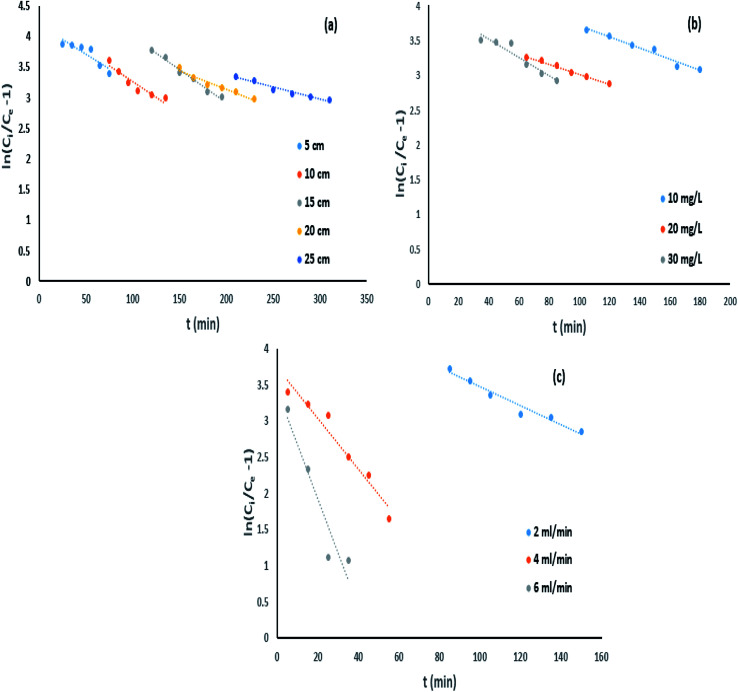
Thomas model impact of (a) column bed height, (b) influent concentration and (c) inflow rate on IBP adsorption.

Thomas model has been established as the most conversant model which is employed to define the static bed column performance for adsorption process.^29^ This model was framed on the basis of both solid–liquid interface mass transfer and chemical interaction between sorbate and sorbent. Thomas model provides the conception of the quantity of movement of sorbate ions from its aqueous phase to the porous exterior of the solid. Hence, linear mode of Thomas model has been signified as eqn (14):^30^14
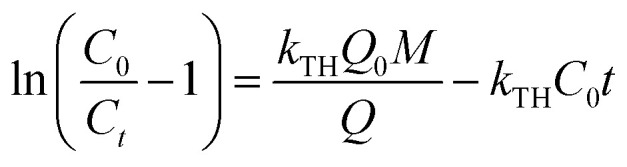
where *k*_TH_ represents the Thomas model constant (L min^−1^ mg^−1^), *Q*_0_ signifies the uptake capacity (mg g^−1^), and *t* denotes total flow time (min). *Q*_0_ and *k*_TH_ were measured from 
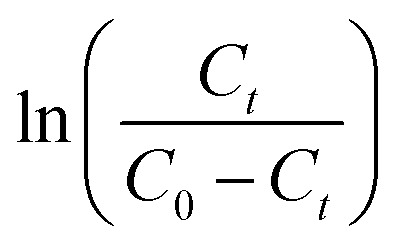
*vs. t* plot (Fig. 10) at a given inflow rate where
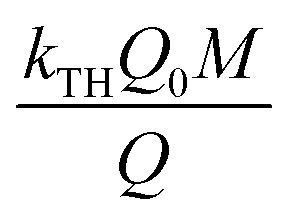
 and *k*_TH_*C*_0_*t* indicates slope and intercept respectively.^31^

Table 5 represents the model parameter outcomes of Thomas model. It was observed that the value of *k*_TH_ along with *Q*_0_ varied with changing column bed length, solution concentration and inflow rate. Table 5 demonstrated the *k*_TH_ value that was noticed to be decreased with rise in bed height of the column that may be due to reduction in sorption rate with rise in bed height whereas influent flow rate and IBP concentration were kept constant. Again, rise in *Q*_0_ value was noticed with increasing column bed length that can be accredited to the matter of maximum opportunity of interaction between sorbate-sorbent. Correspondingly, when inlet IBP concentration was varied the value of *Q*_0_ increased with rise in influent concentration and value of *k*_TH_ decreased with rising influent concentration. When inlet flow rate was varied, it was noticed that with intensification in flow rate the *k*_TH_ value was increased which resulted in less adsorption due to low residence time, and value of *Q*_0_ was decreased with rise in inflow rate. This can be attributed to reduction of retention time of sorption at higher flow rate.

**Table 5:** Model parametric results found from Yoon–Nelson and Thomas model

Parameters			Thomas model			Yoon–Nelson model		
*C*_i_	*Q*	*Z*	*Q*_0_	*k*_TH_	*R* ^2^	*τ*	*k*_YN_	*R* ^2^
10	2	5	3.09	0.097	0.835	136.18	0.0022	0.898
10	2	10	4.27	0.018	0.891	281.65	0.0175	0.997
10	2	15	4.90	0.010	0.983	470	0.0108	0.983
10	2	20	5.38	0.006	0.955	830.4	0.005	0.983
10	2	25	6.52	0.004	0.961	1080.526	0.0038	0.932
10	2	20	30.13	0.081	0.965	683.6508	0.0063	0.984
20	2	20	56.04	0.036	0.987	494.1333	0.005	0.98
30	2	20	59.68	0.029	0.915	218.125	0.0016	0.857
10	2	20	2.68	0.001	0.962	384.5868	0.0121	0.952
10	4	20	1.57	0.003	0.952	92.15859	0.0454	0.979
10	6	30	1.01	0.007	0.911	45.66088	0.0749	0.911

#### 3.8.3. Yoon–Nelson model

**Fig. 11:**
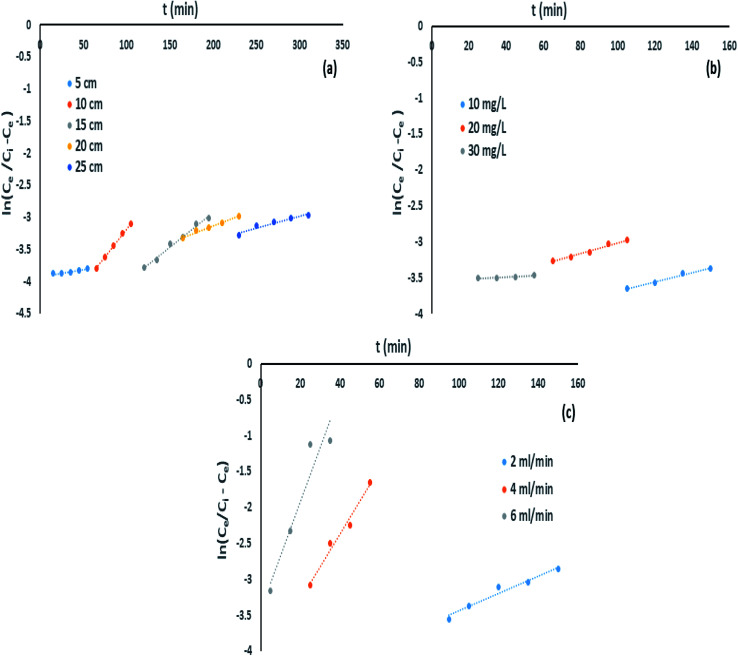
Yoon–Nelson model impact of (a) bed height, (b) initial influent concentration and (c) flow rate on IBP adsorption.

Yoon–Nelson model abridges the enactment of a column sorption with less parametric conditions. This model comprises fewer aspects which shrinks the errors designed from the earlier models. Similarly, this model preferably applies on single sorbate arrangement. Therefore, eqn (15) can be designated as linear forms of Yoon–Nelson model.^32^15
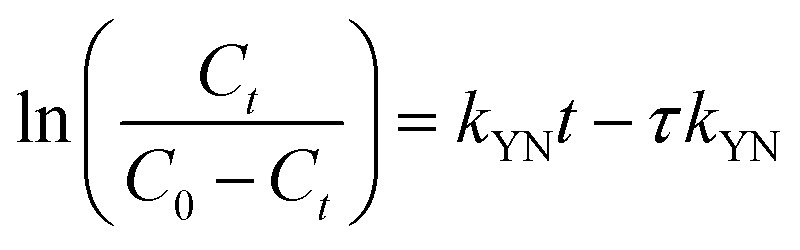
where *k*_YN_ indicates Yoon–Nelson rate velocity constant in L min^−1^ and *τ* implies time needed in min for 50% breakthrough point of sorbate. The data of *k*_YN_ and *τ* was found from slope and intercept of 
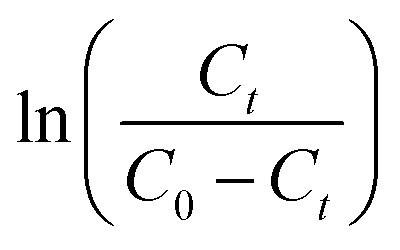
 against *t* plot respectively (Fig. 11).

In the current study, factors of Yoon–Nelson model *k*_YN_ and *τ* were varied with changing the experimental conditions and calculated values were presented in Table 5. The *τ* value was increased with increasing bed height and it was maximum at highest bed height. On the other hand, *τ* value was decreased with decreasing both influent (IBP) concentration and inlet flow rate. It was observed that lowest *τ* value was achieved at maximum flow rate. Once more, *k*_YN_ value was detected maximum at higher flow rate and lowest at higher bed height. These findings can be accredited to the point that at higher retention time greater sorption are possible. Hence, this model recommends from parametric estimation that variation of flow rate and bed depth are significant parameters for fabrication of sorption column at a greater scale.

### 3.9. Column desorption–adsorption cycles

**Fig. 12:**
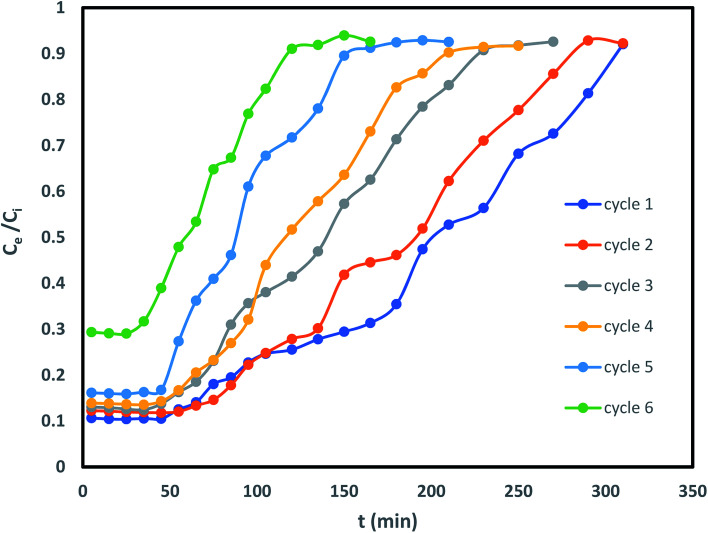
Desorption cycle of IBP exclusion by Zr(DSPB)Al beads.

Column reusability study is an imperative criterion for endorsing its usage on industrial scale. Therefore, IBP laden sorbent beads were regenerated with 0.6 N methanol for desorption and subsequently saturation of the column was occurred with IBP ions. As shown in Fig. 12 and Table 6 that after desorption, the sorbent was capable of detaching IBP moieties from the aqueous solution up to 4 cycles competently through of 55% regeneration percentage; afterward there was a change in the nature of the breakthrough curve. Consequently, desorption breakthrough curve kept its consistency till 5 cycles and 15.128% elution has been found. This may be attributed to the fact that sorbent confined in Zr(iv) allied alginate bead was competent to embrace its sorption–desorption mechanism for higher number of series. This may be explained in the manner that the sorbents had a positive revelation with minor probabilities of overcrowding.

**Table 6:** Performance of column for IBP sorption–desorption cycles by Zr(DSPB)Al beads

Cycle	*q*_total(desorp)_	*q*_eq(desorp)_	EI%	*Q*_total(reg)_	*q*_eq(reg)_	Reg%
1	11.41	5.188	25.235	7.73	3.51	87.35
2	10.77	4.895	24.562	7.24	3.29	81.81
3	9.18	4.172	20.317	5.50	2.50	62.22
4	9.17	4.168	20.474	4.95	2.25	55.94
5	6.65	3.025	15.516	3.32	1.51	37.59
6	4.96	2.254	11.890	2.38	1.08	26.99

### 3.10. Cost estimation of Zr(DSPB)Al beads production

The estimation of sorbent manufacturing cost is significant aspect towards implementation of adsorbent in larger scale approach. In our earlier work DSPB production cost was reported. The cost analysis of Zr(DSPB)Al beads in Indian rupee (INR) has been calculated and revealed in Table 7. An inclusive cost assessment of zirconium caged alginate bead preparation has not been reported yet. The total production price of Zr(DSPB)Al beads was 245.84 INR per kg. Hence, the production cost of Zr(DSPB)Al beads was considered to be moderate and it can be successfully used as promising sorbent towards elimination of IBP in a static bed column.

**Table 7:** Cost analysis of Zr(DSPB)Al beads preparation

Cost analysis of Zr(DSPB)Al beads preparation			
Specifics	Sub segments	Break up of cost	Total cost per kg (INR)
Production of DSPB biochar	Already reported in our earlier work		137.36 [17]
Preparation of Zr(DSPB)Al bead	Sodium alginate gel preparation	Sodium alginate powder	48.75
		Distilled water	5
	Preparation of zirconium (Zr) solution	Zirconium powder cost	19.31
		Distilled water	12.5
Net cost			222.92
100% of overall cost			22.92
Total cost			245.84

## 4. Conclusions

The current investigation accomplishes that the date seed derived steam activated biochar associated with zirconium coupled sodium alginate beads is evidenced to be an encouraging means towards IBP exclusion from water bodies in the static bed column. The exploration revealed that the Zr(DSPB)Al adsorbent produced reasonably favourable result of removal of IBP by 94.86% by maintaining the optimized column bed height of 20 cm, influent concentration 30 mg L^−1^ and inflow rate of 2 mL min^−1^. BDST model, Thomas model and Yoon–Nelson model were analysed to investigate the efficiency of sorption process. BDST model proposed rise in bed height favours better sorption as increase of column EBRT and sorption capability of sorbent. Thomas model and Yoon–Nelson model designated the influence of inflow rate and IBP influent concentration validates the investigational explanations. Regeneration study revealed that the sorbent was capable of sorption ability for multiple cycles (upto 5 cycle). The cost assessment of Zr(DSPB)Al beads was also presented as an effective sorbent for IBP removal study. Therefore, it may be established that at optimal bed height of 20 cm the Zr(DSPB)Al beads could be a perfect sorbent for IBP sorption and can be applied in larger scale operation.

## Conflicts of interest

No conflicts are there to disclose.

## Supplementary Material
